# Predictors of HIV Among 1 Million Clients in High-Risk Male Populations in Tanzania

**DOI:** 10.1007/s10461-022-03667-9

**Published:** 2022-04-01

**Authors:** Gaspar Mbita, Albert N. Komba, Caterina Casalini, Eva Bazant, Kelly Curran, Alice Christensen, Daniel Nyato, Young-Mi Kim, Jason Reed, Neema Makyao, Upendo Kategile, Donaldson F. Conserve, Diana Faini, Jos van Roosmalen, Thomas van den Akker

**Affiliations:** 1 Jhpiego, Dar-es-Salaam, Tanzania; 2 The Task Force for Global Health, Decatur, GA USA; 3 Jhpiego, Baltimore, MD USA; 4 Institute of Population Health Sciences, Queen Mary University of London, London, UK; 5 Ministry of Health Community Development, Gender, Elderly, and Children, National AIDS Control Program, Dodoma, Tanzania; 6 USAID, Dar-es-Salaam, Tanzania; 7 Department of Prevention and Community Health, Milken Institute School of Public Health, George Washington University, Washington, DC USA; 8 Department of Epidemiology and Biostatistics, Muhimbili University of Health and Allied Sciences, Dar-es-Salaam, Tanzania; 9 Athena Institute, Vrije Universiteit, Amsterdam, The Netherlands; 10 Department of Obstetrics and Gynecology, Leiden University Medical Center, Leiden, The Netherlands; 11 Jhpiego, Monrovia, Liberia

**Keywords:** Men who have sex with men, Male client of female sex workers, Men living in areas with high risk of HIV, Sexually transmitted infections, Tanzania

## Abstract

The World Health Organization identified men as an essential group to target with HIV testing and treatment strategies;: men who have sex with men (MSM) and male clients of female sex workers (CFSW) account for 35% of new HIV infections globally. Using a cross-sectional design from a community-based HIV prevention project in Tanzania (October 2015–September 2018) and multivariable logistic regression, we identified predictors of HIV seropositivity among men. Of 1,041,343 men on their initial visit to the project, 36,905 (3.5%) were MSM; 567,005 (54.5%) were CFSW; and 437,343 (42.0%) were other men living near hotspots (OMHA). Three predictors of HIV seropositivity emerged across all three groups: being uncircumcised, having sexually transmitted infection symptoms, and harmful drinking of alcohol before sex. Any reported form of gender-based violence among MSM and OMHA and inconsistent condom use among CFSW were associated with HIV seropositivity. These findings may inform community HIV strategies like self-testing, delivery of pre-exposure prophylaxis and antiretroviral therapy, and behavioral change communication targeting men at higher risk of infection.

## Introduction

At the end of 2019, 38 million people globally were living with HIV. During that year, 1.7 million people were newly infected [1] and one-third of new global HIV infections were among men who have sex with men (MSM) and clients of female sex workers (CFSW) [2].

Due to dramatically lower testing uptake among men, the World Health Organization identified men as a critical group to reach with innovative HIV testing services and enrollment into treatment [3]. In Tanzania, compared to women, men are lagging behind with regard to the 95–95–95 UNAIDS targets. In Tanzania, at the end of 2017, only 75% of male adults, aged ≥ 15, and living with HIV knew their HIV status, compared to 84% of their female counterparts. The proportion of male adults aged ≥ 15 who knew their status and were on antiretroviral therapy was 74% compared to 81% among their female counterparts. However, achievement of viral suppression while on antiretroviral therapy was similar (85% and 87%) among males and females, respectively [4]. Part of the discrepancy between men and women is the opportunity for HIV testing among women during antenatal care. Structural and cultural barriers among men, including men's mobility and gender norms valorizing risk-taking and discouraging health-seeking behavior, affect men's participation in HIV testing relative to women [5, 6].

Globally, the risk of acquiring HIV varies considerably within male subgroups, such as MSM and CFSW. The risk of acquiring HIV is 22 times higher among MSM than men in the general population in Tanzania [4]. Prompt innovative approaches for testing, diagnosis, treatment, and viral suppression among men are required to reduce national HIV incidence rates [7, 8].

Globally, studies across regions have reported several factors associated with HIV infection among men. Evidence shows that HIV infection among MSM is propagated through inconsistent condom use. A study in Benin indicated that MSM who did not consistently use condoms during anal sex with a male sex partner were four times more likely to get infected with HIV than others [9]. Studies conducted in sub-Saharan Africa and Asia reported the association of higher age, unmarried status, and not being circumcised with increasing risks of HIV [10, 11]. However, married men in Zimbabwe had higher rates of HIV infection than unmarried men [12]. Studies in the United States reported that divorced and separated men had higher HIV mortality rates than married men, which may be due to their participation in sex markets or networks, leading to more sexual partners and increasing their risk of HIV infection [13]. Studies conducted in Tanzania and Rwanda indicated that men with sexually transmitted infections (STIs) had higher HIV seroconversion [14, 15]. Studies in South Africa reported lifetime experience of gender-based violence (GBV) is associated with HIV risk acquisition [16–18]. Additionally, in the Southern Highlands of Tanzania and Uganda, an association between alcohol consumption before sex and the risk of HIV infections was reported [11, 19].

Despite literature evaluating risk factors associated with HIV for men [10, 14–18, 20–28], little is known about risk factors for HIV transmission specific to high-risk male subgroups in Tanzania [15]. To curb the rate of new infections in countries with generalized epidemics such as Tanzania and improve service uptake among men, it is imperative to understand seropositivity among male subgroups and factors associated with HIV acquisition.

This study aims to assess factors associated with HIV seropositivity among three groups of men at high risk of HIV transmission seeking HIV services: MSM, CFSW, and other men living in and around areas with high HIV acquisition or “hot spots” (OMHA). To our knowledge, this is the first large-scale analysis conducted in Tanzania that included men at high risk of HIV who were recruited at health service locations.

## Methods

### Study Design, Setting, and Description

**Fig. 1:**
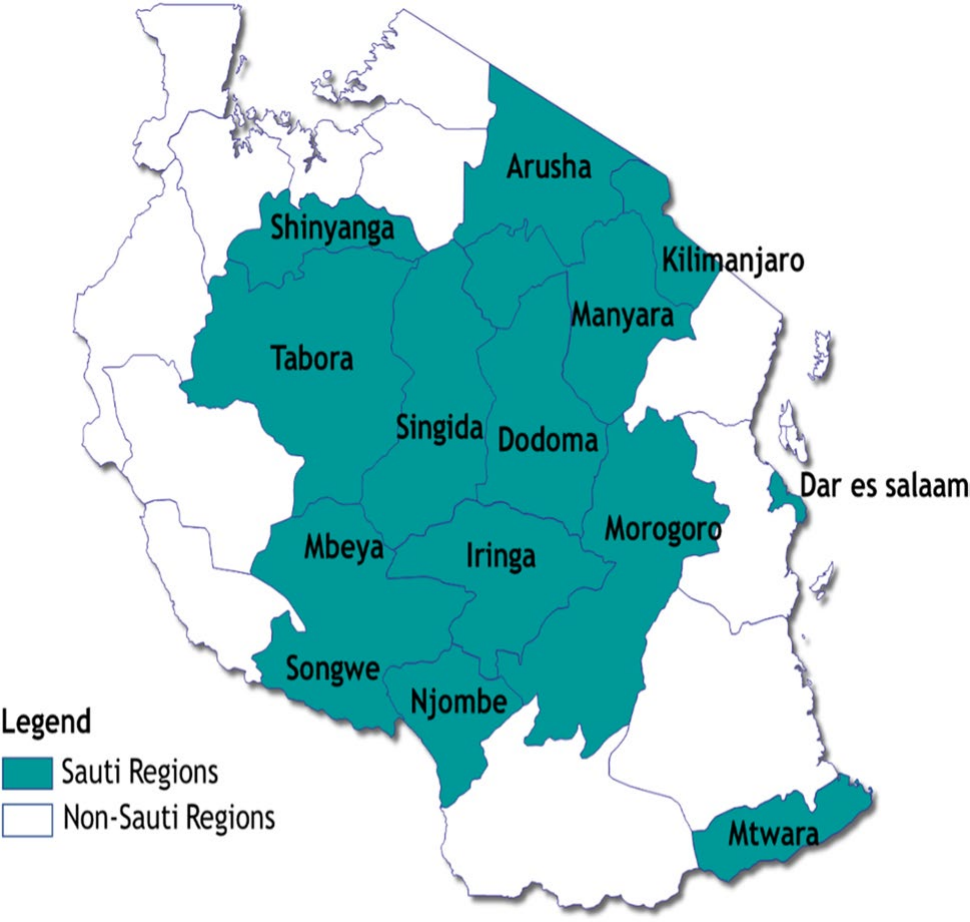
Map of Sauti Project regions included in the analysis

This analysis used secondary data from a cross-sectional study nested within a large-scale community-based HIV prevention program, called the Sauti Project [19, 29, 30], funded by the U.S. President’s Emergency Plan for AIDS Relief/U.S. Agency for International Development, to reach vulnerable and high-risk populations in Tanzania. Jhpiego, an affiliate of Johns Hopkins University, implemented the Sauti Project with its partners EngenderHealth, Inc.; Pact, Inc.; and the National Institute for Medical Research. The project delivered services in 14 of 26 regions of Tanzania mainland between October 2015 and January 2020 (Fig. 1).

The Sauti Project engaged regionally-based mobile nurses who provided clinical services to vulnerable and high-risk populations at hot spots, defined as areas of high HIV transmission. The hotspots include brothels, mining and fishing villages, plantations, truck drivers’ truck stops, and social venues such as bars, nightclubs, and guesthouses. Clinical care included HIV testing; escorted linkage to HIV care and treatment; sexual risk assessment; provision of condoms and family planning services; screening for STIs, tuberculosis (TB), drug abuse, GBV services; and referral to post-GBV services (social, legal, and medical care).

### Study Population

We included all clients who attended Sauti services for the first time between October 2015 and September 2018. In this analysis, the male clients had self-reported the characteristics meeting our project’s definitions of MSM, CFSW, OMHA, and ≥ 18 years. We defined a CFSW as a person self-identifying as male reporting paying for sex with a female in the past 12 months. We described MSM as men who engage in sexual relations with other men, including paying for sex, and OMHA as a person’s self-reporting as male, living in and around areas with high risks of HIV acquisition, and not fitting into a category of MSM or CFSW.

### Data Collection, Management, and Quality Assurance Procedures

Data were collected using paper-based client record forms known as Health Screening and Service Tool (HSST). HSST was a comprehensive tool used to record client-level biomedical services at the first and any follow-up visits. Information collected included: type of visit, client’s socio-demographic data, sexual health and risk behavior assessment; screening for harmful drinking of alcohol and substance use, GBV, and TB; HIV risk screening and testing, STI screening and treatment, oral pre-exposure prophylaxis, and prevention of unwanted pregnancy.

Data from this analysis came from Sauti’s database of individual-level records of its clients. At service delivery points, trained health care providers recorded the client’s information in the HSST during clinical consultations. Providers validated the HSST with the client before the client left the venue. Providers submitted HSSTs to regional offices. At regional Sauti offices, trained data clerks and regional monitoring and evaluation officers, along with providers, checked completeness and verified other validation rules. Then, data clerks entered data into the Sauti database. At the project’s head office (Jhpiego Tanzania office), the informatics and data management team ran data validation queries and cleared them with regional offices after reviewing the source document (HSST). Every month, the data management team examined data entry consistency by randomly selecting 10% of HSSTs recorded HIV-negative clients and 100% of registered HIV-positive clients and counter-verifying against Sauti database records. For this analysis, data from the database were de-identified and imported into Stata, where data cleaning and management took place. Data quality cleaning on Stata involved running validation rules for variables in the semantic areas and checking for missing information through cross-tabulations. The dataset analysis was checked by an external biostatistician from Johns Hopkins University.

### Laboratory Tests and Methods

HIV seropositivity was established with rapid diagnostic tests (RDTs) used by the Ministry of Health, Community Development, Gender, Elderly, and Children to scale up HIV testing. RDTs were performed by trained HIV testing and counseling (HTS) health workers and laboratory professionals. As per Tanzania’s national policy, which permits HIV testing at both facility and community level, data for our study came from clients tested within the community. All participants underwent regular HIV testing using standard algorithms, which included HIV rapid testing using SD Bioline HIV ½ (T1) (Standard Diagnostics Inc., Suwon, Korea) and Uni-Gold Recombigen HIV test (T2) (Trinity Biotech, Wicklow, Ireland). HTS health workers reported samples that were non-reactive on T1 as HIV-negative. Reactive samples on the first test (T1 positive) were tested with a separate and distinct second test (T2) comprised of a different antigen preparation to avoid false cross-reactivity with T1.

HIV seropositive clients were reported in HSSTs by testing positive using standard algorithms, including HIV rapid testing using T1 and T2.

### Statistical Analysis

The main outcome variable was HIV serostatus. HIV seropositivity was calculated by dividing the total number of men with HIV reactive test results by the total number of men who received the HIV test. We selected the potential covariates of HIV seropositivity based on the variables associated with higher HIV positivity reported in the Tanzania HIV Impact Survey 2016–2017 and elsewhere [4, 9, 11–18, 21, 22, 28, 29]. We transformed age from the continuous variable into a categorical variable to analyze the association between age groups and HIV seropositivity. Socio-demographic characteristics included age (age group: 25–34, 35–44, ≥ 45 years compared with 18–24 years); marital status (married/cohabiting, divorced compared with single); education (primary, secondary/higher education compared with never/some primary). We examined sexual risk behaviors: number of sex partners in the past week (more than one compared with one partner); condom use in the past month (inconsistent use compared with consistent use); harmful drinking of alcohol (harmful drinking, not drinking, compared with unharmful drinking). The project used the “alcohol use disorders identification test” developed by the World Health Organization as a simple screening tool to determine if a person's alcohol consumption was harmful [31–33]. Drugs or substance use before sex in the past month (using, not disclosed compared with not using). We examined clinical characteristics: syndromic STIs screening status (having symptoms compared to having no symptoms); men’s circumcision status (not circumcised compared with circumcised); and GBV screening status (experienced any form of violence, not screened compared with not experienced any form of violence). We compared HIV seropositivity across socio-demographic characteristics, sexual risk factors, and characteristics assessed in the clinical examination.

We examined frequencies and percentages of each categorical variable and assessed their relationship to HIV status using Pearson's chi-squared tests (χ^2^). Variables associated with HIV status (p < 0.05) were considered risk factors. To examine the predictors of HIV seropositivity, we conducted both bivariate and multivariable analyses. In the bivariate analyses, we compared clients' socio-demographic characteristics, sexual risk behaviors, and clinical characteristics of men with a reactive test result to men with a non-reactive test result in each group. We constructed a multivariable logistic regression model to examine independent factors associated with HIV infection.

We built a logistic random-effects regression model to assess predictors of HIV seropositivity by accounting for variations between regions of service (clustering effect) to eliminate over- and under-estimation in standard errors. We estimated covariates’ adjusted odds ratios (aOR) with 95% confidence intervals (CI). Socio-demographic, sexual risk behaviors, and clinical characteristics were included in the final multivariable model based on their statistically significant association with HIV seropositivity in bivariate analyses at p < 0.05. Variables not statistically significant in univariate analysis were assessed individually and did not have any substantial effect on odds ratios for the primary association.

All data analysis used Stata software version 15.0 (Stata Statistical Software: Release 15. 2017. College Station, TX: StataCorp LLC.)

### Ethical Oversight

Approval to conduct secondary analysis of Sauti program data was obtained from the institutional review boards of Johns Hopkins Bloomberg School of Public Health (IRB No 00006673) and the National Institute of Medical Research of Tanzania (NIMR/HQ/R.8c/Vol.1/678). A detailed consent script was discussed with each client and each client gave written consent for receipt of care, including HIV testing, follow-up contact for care or contacting partners as needed, and for use of the client’s de-identified data for program improvement.

## Results

### Baseline Characteristics of Study Participants

This analysis included 1,041,343 men who made an initial visit at Sauti Project outreach services from October 2015 to September 2018. Of these, 36,905 (3.5%) were MSM, 567,005 (54.5%) CFSW, and 437,343 (42.0%) were OMHA (Table 1). The median age for MSM was 26.6 years (interquartile range [IQR] 24.0–33.3), 30.0 years (IQR 26.0–36.0) for CFSW, and 32.0 years (IQR 27.8–38.0) for OMHA.

**Table 1:** Characteristics of men accessing Sauti services

Variable	Population category of men					
	MSM		CFSW		OMHA	
	N	%	N	%	N	%
Total (row %)	36,905	3.5	567,005	54.5	437,343	42.0
Age (years)						
Median (IQR)	26.6 (24.0–33.3)		30.0 (26.0–36.0)		32.0 (27.8–38.0)	
18–24	10,858	29.4	107,810	19.0	72,940	16.7
25–34	17,979	48.7	300,834	53.1	225,363	51.5
35–44	5574	15.1	107,770	19.0	70,169	16.0
45 +	2494	6.8	50,591	8.9	68,871	15.8
Marital status						
Single	18,366	49.8	215,531	38.0	206,402	47.2
Married/cohabiting	16,682	45.2	317,504	56.0	210,720	48.2
Divorced	1857	5.0	33,970	6.0	20,221	4.6
Education						
Never/some primary	3,878	10.5	40,493	7.1	77,658	17.8
Primary education	12,281	33.3	154,369	27.2	89,883	20.6
Secondary/Higher education	20,746	56.2	372,143	65.6	269,802	61.7
Condom use						
Consistent use	4300	11.7	48,512	8.6	8628	2.0
Inconsistent use	32,605	88.4	518,493	91.4	428,715	98.0
Circumcision						
Yes	15,000	40.6	333,919	58.9	241,987	55.3
No	21,905	59.4	233,086	41.1	195,356	44.7
Syndromic STI						
No symptoms	29,504	80.0	447,436	78.9	332,405	76.0
Having symptoms	530	1.4	2362	0.4	1560	0.4
Not screened	6871	18.6	117,207	20.7	103,378	23.6
Number of sexual partners						
Having one partner	17,698	48.0	337,834	59.6	334,355	76.5
Having more than one partner	19,207	52.0	229,171	40.4	102,988	23.6
Harmful drinking of alcohol before sex						
Not harmful	28,585	77.5	495,072	87.3	413,710	94.6
Harmful	7046	19.1	63,222	11.2	17,055	3.9
Not using	1274	3.5	8711	1.5	6578	1.5
Use drug or substance during sex in last month						
Not using	31,936	86.5	546,628	96.4	428,712	98.0
Using	3877	10.5	11,946	2.1	3749	0.9
Not disclosed	1092	3.0	8431	1.5	4882	1.1
Reported any form of GBV						
Not reported	26,437	71.6	419,788	74.0	290,648	66.5
Reported	572	1.6	3977	0.7	2143	0.5
Not screened	9896	26.8	143,240	25.3	144,552	33.1

*MSM* men who have sex with men; *CFSW* clients of female sex workers; *OMHA* other men living in and around areas with high HIV acquisition; *IQR* interquartile range; *STI* sexually transmitted infection; *GBV* gender-based violence

Among MSM, 18,539 (50.2%) were either married/cohabiting or divorced, 19,207 (52.0%) had at least two partners, and 32,605 (88.4%) reported using condoms inconsistently. Among CFSW, 518,493 (91.4%) reported using condoms inconsistently and among OMHA, 428,715 (98.0%) reported inconsistent condom use in the past month (Table 1).

### HIV Seropositivity Among MSM, CFSW, and OMHA

The HIV seropositivity among MSM was 4.1% and unadjusted analysis indicated statistically significant association by HIV seropositivity for condom use (χ^2^ = 15.9, degrees of freedom [df] = 1, p < 0.001), syndromic STI (χ^2^ = 168.5, df = 2, p < 0.001), number of sexual partners (χ^2^ = 108.1, df = 1, p < 0.001), harmful drinking of alcohol before sex (χ^2^ = 86.2, df = 2, p < 0.001), and reported any form of GBV (χ^2^ = 122.3, df = 2, p < 0.001) (Table 2).

**Table 2:** HIV seropositivity among MSM

Variables	Total tested	Tested HIV− N (%)	Tested HIV+ N (%)	Chi-squared test statistics	Degree of freedom	p-value
HIV status						
Positive	36,905	35,378 (95.9)	1527 (4.1)			
Age (years)						
18–24	10,858	10,502 (96.7)	356 (3.3)	61.5	3	< 0.001
25–34	17,979	17,249 (95.9)	730 (4.1)			
35–44	5574	5288 (94.9)	286 (5.1)			
45 +	2494	2339 (93.8)	155 (6.2)			
Marital status						
Single	18,366	17,575 (95.7)	791 (4.3)	81.5	2	< 0.001
Married/cohabiting	16,682	16,092 (96.5)	590 (3.5)			
Divorced	1,857	1,711 (92.1)	146 (7.9)			
Education						
Never/some primary	3878	3717 (95.8)	161 (4.2)	85.7	2	< 0.001
Primary education	12,281	11,935 (97.2)	346 (2.8)			
Secondary/Higher education	20,746	19,726 (95.1)	1020 (4.9)			
Condom use						
Consistent use	4300	4171 (97.0)	129 (3.0)	15.9	1	< 0.001
Inconsistent use	32,605	31,207 (95.7)	1398 (4.3)			
Circumcision						
Yes	15,000	14,557 (97.1)	443 (3.0)	89.4	1	< 0.001
No	21,905	20,821 (95.0)	1084 (5.0)			
Syndromic STI						
No symptoms	29,504	28,336 (96.0)	1168 (4.0)	168.5	2	< 0.001
Having symptoms	530	449 (84.7)	81 (15.3)			
Not screened	6871	6593 (96.0)	278 (4.0)			
Number of sexual partners						
Having one partner	17,698	16,767 (94.7)	931 (5.3)	108.1	1	< 0.001
Having more than one partner	19,207	18,611 (96.9)	596 (3.1)			
Harmful drinking of alcohol before sex						
Not harmful	28,585	27,543 (96.4)	1042 (3.6)	86.2	2	< 0.001
Harmful	7046	6,616 (93.9)	430 (6.1)			
Not using	1274	1219 (95.7)	55 (4.3)			
Use drug or substance during sex in last month						
Not using	31,936	30,698 (96.1)	1238 (3.9)	50.9	2	< 0.001
Using	3877	3670 (94.7)	207 (5.3)			
Not disclosed	1092	1010 (92.5)	82 (7.5)			
Reported any form of GBV						
Not reported	26,437	25,430 (96.2)	1007 (3.8)	122.3	2	< 0.001
Reported	572	498 (87.1)	74 (12.9)			
Not screened	9896	9450 (95.5)	446 (4.5)			

*MSM* men who have sex with men, *CFSW* clients of female sex workers; *OMHA* other men living in and around areas with high HIV acquisition; *STI* sexually transmitted infection; *GBV* gender-based violence

Among CFSW, the HIV seropositivity among MSM was 3.6% and unadjusted analysis indicated statistically significant association by HIV seropositivity for condom use (χ^2^ = 495.5, df = 1, p < 0.001), syndromic STI (χ^2^ = 556.7, df = 2, p < 0.001), number of sexual partners (χ^2^ = 649.1, df = 1, p < 0.001), and harmful drinking of alcohol before sex (χ^2^ = 309.2, df = 2, p < 0.001) (Table 3).

**Table 3:** HIV seropositivity among CFSW

Variables	Total tested	Tested HIV+ N (%)	Tested HIV+ N (%)	Chi-squared test statistics	Degree of freedom	p-value
HIV status						
Positive	567,005	547,195 (96.4)	19,810 (3.6)			
Age (years)						
18–24	107,810	106,483 (98.8)	1327 (1.2)	3500	3	< 0.001
25–34	300,834	290,934 (96.7)	9900 (3.3)			
35–44	107,770	102,298 (94.9)	5472 (5.1)			
45 +	50,591	47,480 (93.9)	3111 (6.1)			
Marital status						
Single	215,531	210,450 (97.6)	5081 (2.4)	3900	2	< 0.001
Married/cohabiting	317,504	305,828 (96.3)	11,676 (3.7)			
Divorced	33,970	30,917 (91.0)	3,053 (9.0)			
Education						
Never/Some primary	40,493	38,960 (96.2)	1533 (3.8)	302	2	< 0.001
Primary education	154,369	150,046 (97.2)	4323 (2.8)			
Secondary/Higher education	372,143	358,189 (96.3)	13,954 (3.7)			
Condom use						
Consistent use	48,512	47,678 (98.3)	834 (1.7)	495.5	1	< 0.001
Inconsistent use	518,493	499,517 (96.3)	18,976 (3.7)			
Circumcision						
Yes	333,919	324,723 (97.2)	9196 (2.8)	1300	1	< 0.001
No	233,086	222,472 (95.4)	10,614 (4.6)			
Syndromic STI						
No symptoms	447,436	433,110 (96.8)	14,326 (3.2)	556.7	2	< 0.001
Having symptoms	2362	2214 (93.7)	148 (6.3)			
Not screened	117,207	111,871 (95.4)	5336 (4.6)			
Number of sexual partners						
Having one partner	337,834	324,302 (96.0)	13,532 (4.0)	649.1	1	< 0.001
Having more than one partner	229,171	222,893 (97.3)	6278 (2.7)			
Harmful drinking of alcohol before sex						
Not harmful	495,072	478,533 (96.7)	16,539 (3.3)	309.2	2	< 0.001
Harmful	63,222	60,248 (95.3)	2974 (4.7)			
Not using	8711	8414 (96.6)	297 (3.4)			
Use drug or substance during sex in last month						
Not using	546,628	527,607 (96.5)	19,021 (3.5)	8.9	2	0.011
Using	11,946	11,484 (96.1)	462 (3.9)			
Not disclosed	8431	8104 (96.1)	327 (3.9)			
Reported any form of GBV						
Not reported	419,788	405,893 (96.7)	13,895 (3.3)	122.3	2	< 0.001
Reported	3977	3786 (95.2)	191 (4.8)			
Not screened	143,240	137,516 (96.0)	5724 (4.0)			

*MSM* men who have sex with men, *CFSW* clients of female sex workers; *OMHA* other men living in and around areas with high HIV acquisition; *STI* sexually transmitted infection; *GBV* gender-based violence

In OMHA, the HIV seropositivity among MSM was 2.4% and unadjusted analysis indicated statistically significant association by HIV seropositivity for condom use (χ^2^ = 243.6, df = 1, p < 0.001), syndromic STI (χ^2^ = 301.9, df = 2, p < 0.001), number of sexual partners (χ^2^ = 149.0, df = 1, p < 0.001), harmful drinking of alcohol before sex (χ^2^ = 483.6, df = 2, p < 0.001) (Table 4).

**Table 4:** HIV seropositivity among OMHA

Variables	Total tested	Tested HIV+ N (%)	Tested HIV+ N (%)	Chi-squared test statistics	Degree of freedom	p-value
HIV status						
Positive	437,343	426,939 (97.6)	10,404 (2.4)			
Age (years)						
18–24	72,940	72,204 (99.0)	736 (1.0)	2400	3	< 0.001
25–34	225,363	221,243 (98.2)	4120 (1.8)			
35–44	70,169	67,383 (96.0)	2786 (4.0)			
45 +	68,871	66,109 (96.0)	2762 (4.0)			
Marital status						
Single	206,402	203,596 (98.6)	2806 (1.4)	2800	2	< 0.001
Married/cohabiting	210,720	204,460 (97.0)	6260 (3.0)			
Divorced	20,221	18,883 (93.4)	1338 (6.6)			
Education						
Never/Some primary	77,658	76,578 (98.6)	1080 (1.4)	450.0	2	< 0.001
Primary education	89,883	87,265 (97.1)	2618 (2.9)			
Secondary/Higher education	269,802	263,096 (97.5)	6706 (2.5)			
Condom use						
Consistent use	8628	8204 (95.1)	424 (4.9)	243.6	1	< 0.001
Inconsistent use	428,715	418,735 (97.7)	9980 (2.3)			
Circumcision						
Yes	241,987	237,674 (98.2)	4313 (1.8)	830.2	1	< 0.001
No	195,356	189,265 (96.9)	6091 (3.1)			
Syndromic STI						
No symptoms	332,405	325,037 (97.8)	7368 (2.2)	301.9	2	< 0.001
Having symptoms	1560	1443 (92.5)	117 (7.5)			
Not screened	103,378	100,459 (97.2)	2919 (2.8)			
Number of sexual partners						
Having one partner	334,355	326,923 (97.8)	7432 (2.2)	149.0	1	< 0.001
Having more than one partner	102,988	100,016 (97.1)	2972 (2.9)			
Harmful drinking of alcohol before sex						
Not harmful	413,710	404,306 (97.7)	9404 (2.3)	483.6	2	< 0.001
Harmful	17,055	16,221 (95.1)	834 (4.9)			
Not using	6578	6412 (97.5)	166 (2.5)			
Use drug or substance during sex in last month						
Not using	428,712	418,581 (97.6)	10,131 (2.4)	35.4	2	< 0.001
Using	3749	3606 (96.2)	143 (3.8)			
Not disclosed	4882	4752 (97.3)	130 (2.7)			
Reported any form of GBV						
Not reported	290,648	284,327 (97.8)	6321 (2.2)	209.2	2	< 0.001
Reported	2143	2032 (94.8)	111 (5.2)			
Not screened	144,552	140,580 (97.3)	3972 (2.7)			

*MSM* men who have sex with men, *CFSW* clients of female sex workers; *OMHA* other men living in and around areas with high HIV acquisition; *STI* sexually transmitted infection; *GBV* gender-based violence

### Predictors of HIV Seropositivity Among High-Risk Men

Controlling for age, marital status, and education, predictors of HIV seropositivity were being uncircumcised (aOR 1.8, 95% CI 1.4–2.3 for MSM; aOR 1.5, 95% CI 1.3–1.8 for CFSWs; and aOR 1.6; 95% CI 1.2–2.2 for OMHA), having STI symptoms (aOR 3.0, 95% CI 2.0–4.5 for MSM; aOR 1.6, 95% CI 1.1–2.4 for CFSWs; and aOR 2.6, 95% CI 2.1–3.2 for OMHA), harmful drinking of alcohol before sex (aOR 1.6, 95% CI 1.2–2.0 for MSM; aOR 1.2, 95% CI 1.1–1.3 for CFSWs; and aOR 1.5, 95% CI 1.2–1.9 for OMHA), reported any form of GBV (aOR 2.0, 95% CI 1.3–2.9 for MSM; and aOR 2.8, 95% CI 1.5–5.1 for OMHA), and inconsistent condom use among CFSW (aOR 1.7, 95% CI 1.3–2.2) (Tables 5, 6, 7).

**Table 5:** Predictors of HIV seropositivity among MSM (n = 36,905)

Variable	HIV+ (%)	Crude odds ratio [95% CI]	Adjusted odds ratio [95% CI]
All (MSM)	4.1		
Age (years)			
18–24	1.3	1	1
25–34	2.7	1.3[1.1–1.4]	1.9[1.7–2.2]
35–44	4.7	1.6[1.4–1.9]	3.2[2.6–4.0]
45 +	4.9	2.0[1.6–2.4]	2.9[1.8–4.7]
Marital status			
Single	4.3	1	1
Married/cohabiting	3.5	0.8[0.7–0.9]	1.4[1.1–1.8]
Divorced	7.9	1.9[1.6–2.3]	3.0[2.6–3.4]
Education			
Never/some primary	4.2	1	1
Primary education	2.8	0.7[0.6–0.8]	1.6[1.3–2.1]
Secondary/Higher education	4.9	1.2[1.0–1.4]	1.3[0.9–1.7]
Condom use			
Consistent use	3.0	1	1
Inconsistent use	4.3	1.5[1.2–1.7]	0.5[0.3–1.1]
Circumcision			
Yes	3.0	1	1
No	4.9	1.7[1.5–1.9]	1.8[1.4–2.3]
Syndromic STI			
No symptoms	4.0	1	1
Having symptoms	15.3	4.4[3.4–5.6]	3.0[2.0–4.5]
Not screened	4.0	1.0[0.9–1.2]	1.3[0.9–1.7]
Number of sexual partners			
Having one partner	5.3	1	1
Having more than one partner	3.1	0.6[0.5–0.6]	1.4[0.9–2.0]
Harmful drinking of alcohol before sex			
Not harmful	3.6	1	1
Harmful	6.1	1.7[1.5–1.9]	1.6[1.2–2.0]
Not using	4.3	1.2[0.9–1.6]	0.9[0.6–1.5]
Use drug or substance during sex in last month			
Not using	3.9	1	1
Using	5.3	1.4[1.2–1.6]	1.1[0.9–1.3]
Not disclosed	7.5	2.0[1.6–2.5]	1.0[0.6–1.9]
Reported any form of GBV			
Not reported	3.8	1	1
Reported	12.9	3.8[2.9–4.8]	2.0[1.3–2.9]
Not screened	4.5	1.2[1.1–1.3]	1.0[0.9–1.3]

MSM, men who have sex with men; CI, confidence interval, STI, sexually transmitted infection; GBV, gender-based violence

**Table 6:** Predictors of HIV seropositivity among CFSW (n = 567,005)

Variable	HIV+ (%)	Crude odds ratio [95% CI]	Adjusted odds ratio [95% CI]
All (CFSW)	3.5		
Age (years)			
18–24	1.2	1	1
25–34	3.3	2.7[2.6–2.9]	2.4[2.1–2.9]
35–44	5.1	4.3[4.0–4.6]	3.6[3.3–3.9]
45 +	6.1	5.3[4.9–5.6]	4.0[3.3–5.0]
Marital status			
Single	2.4	1	1
Married/cohabiting	3.7	1.6[1.5–1.6]	1.2[0.9–1.4]
Divorced	9.0	4.1[3.9–4.3]	2.6[2.1–3.3]
Education			
Never/some primary	3.8	1	1
Primary education	2.8	0.7[0.7–0.8]	0.9[0.7–1.0]
Secondary/Higher education	3.7	0.9[0.9–1.0]	0.7[0.5–0.9]
Condom use			
Consistent use	1.7	1	1
Inconsistent use	3.7	2.1[2.0–2.3]	1.7[1.3–2.2]
Circumcision			
Yes	2.8	1	1
No	4.6	1.7[1.6–1.7]	1.5[1.3–1.8]
Syndromic STI			
No symptoms	3.2	1	1
Having symptoms	6.3	2.0[1.7–2.4]	1.6[1.1–2.4]
Not screened	4.6	1.4[1.4–1.5]	1.6[1.2–2.1]
Number of sexual partners			
Having one partner	4.0	1	1
Having more than one partner	2.7	0.7[0.6–0.7]	0.8[0.6–0.9]
Harmful drinking of alcohol before sex			
Not harmful	3.3	1	1
Harmful	4.7	1.4[1.4–1.5]	1.2[1.1–1.3]
Not using	3.4	1.0[0.9–1.1]	0.9[0.7–1.3]
Use drug or substance during sex in last month			
Not using	3.5	1	1
Using	3.9	1.1[1.1–1.2]	0.9[0.8–1.1]
Not disclosed	3.9	1.1[1.1–1.3]	0.9[0.8–1.2]
Reported any form of GBV			
Not reported	3.3	1	1
Reported	4.8	1.5[1.3–1.7]	1.3[0.7–2.2]
Not screened	4.0	1.2[1.2–1.3]	0.9[0.9–1.1]

*CFSW* clients of female sex workers; *CI* confidence interval; *STI* sexually transmitted infection; *GBV* gender-based violence

**Table 7:** Predictors of HIV seropositivity among OMHA (n = 437,343)

Variable	HIV+ (%)	Crude odds ratio [95% CI]	Adjusted odds ratio [95% CI]
All (OMHA)	2.4		
Age (years)			
18–24	1.0	1	1
25–34	1.8	1.8[1.7–2.0]	1.4[1.1–1.8]
35–44	4.0	4.1[3.7–4.4]	1.8[1.2–2.7]
45 +	4.0	4.1[3.8–4.5]	2.2[1.5–3.2]
Marital status			
Single	1.4	1	1
Married/cohabiting	3.0	2.2[2.1–2.3]	0.7[0.6–0.9]
Divorced	6.6	5.1[4.8–5.5]	1.4[1.1–1.7]
Education			
Never/Some primary	1.4	1	1
Primary education	2.9	2.1[2.0–2.3]	0.9[0.5–1.5]
Secondary/Higher education	2.5	1.8[1.7–1.9]	1.0[0.6–1.8]
Condom use			
Consistent use	4.9	1	1
Inconsistent use	2.3	0.5[0.4–0.5]	1.1[0.9–1.3]
Circumcision			
Yes	1.8	1	1
No	3.1	1.8[1.7–1.8]	1.6[1.2–2.2]
Syndromic STI			
No symptoms	2.2	1	1
Having symptoms	7.5	3.6[3.0–4.3]	2.6[2.1–3.2]
Not screened	2.8	1.3[1.2–1.3]	0.9[0.5–1.6]
Number of sexual partners			
Having one partner	2.2	1	1
Having more than one partner	2.9	1.3[1.3–1.4]	0.8[0.6–1.1]
Harmful drinking of alcohol before sex			
Not harmful	2.3	1	1
Harmful	4.9	2.2[2.1–2.4]	1.5[1.2–1.9]
Not using	2.5	1.1[1.0–1.3]	1.1[0.6–2.1]
Use drug or substance during sex in last month			
Not using	2.4	1	1
Using	3.8	1.6[1.3–1.9]	1.1[0.5–2.2]
Not disclosed	2.7	1.1[0.9–1.4]	1.4[0.8–2.4]
Reported any form of GBV			
Not reported	2.2	1	1
Reported	5.2	2.5[2.0–3.0]	2.8[1.5–5.1]
Not screened	2.7	1.3[1.2–1.3]	1.3[0.7–2.4]

*OMHA* other men living in and around areas with high HIV acquisition; *CI* confidence interval; *STI* sexually transmitted infection; *GBV* gender-based violence

## Discussion

This study is the first extensive analysis of more than 1 million initial HIV-care visits among key male populations in Tanzania. It describes three subgroups of men at high risk of HIV acquisition. The data in this analysis indicated higher HIV seropositivity among MSM and CFSW than men in the general population in Tanzania, as seen in other countries [4, 34]. The male populations in this analysis have a high frequency of partner shift along with the high HIV prevalence [35]. Studies have reported that members of key and vulnerable populations, including MSM, CFSW, and OMHA, act as drivers of the HIV epidemic globally [34]. Therefore, it is more likely that these groups could contribute to the epidemic in Tanzania. Predictors of HIV seropositivity across MSM, CFSW, and OMHA include not being circumcised, having STI symptoms, being exposed to any form of GBV, and harmful drinking of alcohol before sex. Understanding the characteristics associated with a positive HIV status can help programs focus their efforts.

Not being circumcised was associated with increased risk of HIV in MSM, CFSW, and OMHA in our study; a systematic review of literature also reported this association for heterosexual and homosexual men [36]. The mechanism supporting the relationship between lack of circumcision and increasing risk of HIV has been reported by previous papers [37, 38]. Circumcision reduces risks of other STI, which in turn reduces the risk of HIV acquisition for males [26, 36]. Prevention interventions, including high coverage of voluntary medical male circumcision, especially for high-risk groups of individuals such as MSM, CFSW, and OMHA, remain important interventions for HIV control.

Our findings suggest that all three groups of men at high risk of HIV with STI symptoms had dramatically higher HIV seroconversion than those without STI symptoms. This is consistent with other studies in sub-Saharan Africa among MSM [14, 15]. STIs mediate local inflammatory responses that increase HIV risks, stressing the importance of providing holistic health care that offers HIV and STI screening and treatment among high-risk men [39, 40].

Previous studies have also confirmed the association of lifetime GBV and HIV seropositivity among MSM [16–18]. Perpetrators of GBV may be more likely to have HIV and impose risky sexual practices on clients and partners, which warrants further exploration. Policies, interventions, and programs for HIV prevention must focus on identifying men at risk for GBV and linking them to protection and assistance services, such as medical, social, and legal care.

Another important finding is the association between HIV seropositivity and harmful drinking of alcohol. This is consistent with a study in the Southern Highland region of Tanzania and Uganda [11, 28]. Use of alcohol lessens perceptions of, and increases exposure to, risky sexual behavior; violence; forced, transactional, and unprotected sex; and rape [28, 41]. Studies in Tanzania and elsewhere reported that people who consume alcohol regularly in places such as bars, local breweries, restaurants, and guesthouses, where they also encounter sex partners, are likely to engage in sexual intercourse under the influence of alcohol [20, 42]. This underscores the importance of integrating messages related to HIV and condom use and other mitigation measures at venues where alcohol is consumed.

The national guideline for comprehensive HIV prevention and treatment interventions for key and vulnerable populations in Tanzania includes community-based outreach efforts in hotspots to reach these populations and connect them to health and other social services. The guideline recommended that programs use a HIV combination prevention package of biomedical, behavioral, and structural approaches that ensure that the confidentiality of individuals' identities are protected and prevent further stigma and discrimination of key populations. The package includes HIV testing, family planning, comprehensive condom programming, targeted social and behavior change communication, antiretroviral therapy, tuberculosis, STI screening and treatment, and voluntary medical male circumcision mainstreamed with GBV prevention. However, additional services are needed for men whose alcohol and substance use is harmful. Based on our findings, HIV prevention policies and guidelines should incorporate access to psychosocial interventions including assessment, counseling, and linkage to rehabilitation services for men whose alcohol and substance use is harmful.

## Strengths and Limitations

This analysis was implemented in the context of a comprehensive, community-based HIV program (real-life program data) with a large sample size of over 1 million records of high-risk men. Data used in this analysis were selected because of confidence in its completeness and quality due to extensive data cleaning and quality assessments during data collection, entry, and analysis. Several findings were observed among all three groups of men at high risk of HIV, such as association with STI symptoms, being uncircumcised, and harmful drinking of alcohol before sex.

In a cross-sectional study, it is not possible to draw conclusions with regard to causality. It is challenging to ascertain the time sequence of whether HIV infection preceded a risk factor or whether the observed associations are the predisposing factors associated with both HIV and risk factors. It is also noted that Tanzanian laws do not recognize commercial sex work or same sex sexual behavior. The program definition of OMHA based on self-reporting from clients during initial clinical visit might misclassify OMHA and therefore overestimate their risk behaviors due to social desirability bias among clients who do not want the community to refer to them as MSM or CFSW. We collected data on MSM, CFSW, and OMHA, but some members of these subgroups may not have attended the project and may not have been included in the analysis. However, with the large sample size and richness of the dataset, these findings may be valuable in identifying the predictors of HIV seropositivity among high-risk men in Tanzania. Interventions to reduce HIV risks in these populations, such as promoting and supplying condoms, screening and treating STIs, HIV testing, pre-exposure prophylaxis, and early treatment initiation, are essential to prevent HIV transmission. We recommend future studies to explore in depth who is represented among OMHA and to investigate the difference between their potential risk behaviors and MSM and CFSW.

## Conclusion

Service statistics data have merit and utility for routine program monitoring and designing informed policies and strategies for adaptation at national and sub-national levels. This paper represents one of the most comprehensive analyses based on more than 1 million records of high-risk men in Tanzania, collected in a real-world care delivery setting. Governments and donors can use these findings to design combined interventions, such as community-based HIV self-testing, pre-exposure prophylaxis, community antiretroviral therapy, and behavioral change communication services that focus on men, such as MSM, CFSW, and OMHA, who at high risk of HIV acquisition to achieve UNAIDS’ goal of 95–95–95 for all populations.

## Data Availability

De-identified data may be made available to individual researchers upon request.

## Code Availability

The codebook associated with this analysis can be made available to individual researchers upon request.
